# Negotiating biophysical limits in the European Union’s bioeconomy: a critical analysis of two conflicts over regulating biomass use in EU policy

**DOI:** 10.1007/s11625-024-01543-0

**Published:** 2024-08-14

**Authors:** Benjamin Fleischmann, Andreas Mayer, Christoph Görg, Melanie Pichler

**Affiliations:** 1 Institute of Social Ecology, Department of Economics and Social Sciences, BOKU University, Schottenfeldgasse 29, 1070 Vienna, Austria; 2 Department of Geography, University of Innsbruck, Innrain 52f, 6020 Innsbruck, Austria

**Keywords:** Bioeconomy, Biophysical limits, Bio-based plastics, Cascading use, Critical policy analysis, Land use competition

## Abstract

The bioeconomy seeks to replace fossil fuels with biomass in various products and industrial sectors. The dominant political bioeconomy project focuses on economic growth and aims to increase biomass demand in the EU. This can exacerbate global land use competition and pressures on ecosystems. However, this project does not consider reducing resource use to tackle biophysical limits. Technological innovations are the means for ensuring sustainability. Few social scientific studies have investigated how actors reproduce or question the dominant bioeconomy project. We contribute to this research gap by using critical policy analysis. We explore how actors address biophysical limits and assert their positions and strategies in policy conflicts stemming from the EU bioeconomy strategy. We thereby identified two central conflicts: regulating bio-based plastics and the cascading use of biomass. Our analysis included position papers, policy documents, and expert interviews. We grouped the actors based on their positions and strategies into three political bioeconomy projects. Thus, in addition to the dominant growth-oriented project, we identified a circular and sufficiency-oriented one. Our analysis indicates that these alternative projects influenced bio-based plastics and bioenergy policies to acknowledge biophysical limits. EU policy even provides measures to reduce plastic use. Nevertheless, the Renewable Energy Directive's approach to cascading use reflects a compromise with the growth-oriented project that might not cap using primary biomass for energy. Overall, we demonstrate that there are potential alliances in promoting alternatives to the dominant bioeconomy project. Setting clear limits is constrained by powerful interests advocating for a growth-oriented bioeconomy.

**Supplementary Information:**

The online version contains supplementary material available at 10.1007/s11625-024-01543-0.

## Introduction

The political project of bioeconomy seeks to replace fossil fuels in various industrial processes, products, and applications (European Commission 2018a). This project “is meant to bring about a particular set of political–institutional changes that will shape the parameters of possible future action.” (Goven and Pavone 2014, p. 6) The definition, scale, and strategic orientation of this project are still debated among science, politics, industry, and civil society (Allain et al. 2022; Bugge et al. 2016; Backhouse et al. 2017). One definition of this project has become dominant in the EU bioeconomy strategy (European Commission 2018a; Bugge et al. 2016). This definition “portrays the large-scale substitution of fossil and mineral resources by biomass-based materials as a feasible goal that will facilitate the transition to a sustainable society” (Boyer et al. 2022). The bioeconomy should ensure continuous economic growth, industry competitiveness, and the creation of new “green” jobs (Eversberg et al. 2023).To enable this growth-oriented project, the EU needs to increase its demand for biomass (Scarlat et al. 2015). However, global land use for food, feed, materials, and energy production already puts high pressure on ecosystems and biodiversity (IPBES 2019). A growing substitution of energy and materials with biomass in the EU increases this pressure (Muscat et al. 2021; O’Brien et al. 2015) and runs the risk of exceeding biophysical limits, i.e., the amount of biomass that can be harvested and mobilized without surpassing the reproductive capacity of land and ecosystems (Erb and Gingrich 2022; Haberl et al. 2007).

Still, the bioeconomy project does not aim to change or limit resource use substantially. Potential future technological advancements and adequate management of private companies are the means for developing the bioeconomy and ensuring its sustainability (Boyer et al. 2022). This strategic orientation conceals material interests behind this project and largely excludes a democratic negotiation of the bioeconomy (Ramcilovic-Suominen 2022a, b). At the same time, the role of societal conflicts in reproducing or contesting the bioeconomy is underexplored in political science research (Eversberg et al. 2023; Böcher et al. 2020). For instance, most studies focus primarily on governance and management challenges for developing the bioeconomy (Devaney et al. 2017; Mustalahti 2018; Schütte 2018). Since the beginning of the last decade, the EU and its Member States have published an increasing number of bioeconomy strategies (European Commission 2022b). A few studies indicate that actors from academia, bio-based industries, industrial agriculture, and forestry shaped these strategies to prioritize economic growth and competitiveness (Hausknost et al. 2017; Lühmann 2020). Nevertheless, the updated EU bioeconomy strategy from 2018 pays more attention to biophysical limits than its predecessor (European Commission 2018a; 2012). Hence, the European Commission (EC)’s recent bioeconomy progress report calls for a more holistic industrial and research policy to enable a sustainable bioeconomy (European Commission 2022b). It highlights that “consumption patterns need to become more sustainable to guarantee environmental integrity, as technological solutions alone cannot close the gap between sustainable supply of biological resources and demand.” (ibid., p. 26) To close this gap, the EC encourages better monitoring, innovation, and technology to optimize resource utilization and adopt circular approaches that enhance biomass availability (European Commission 2018a; 2022b). Apart from these primarily technical improvements of resource efficiency, the current bioeconomy strategy does not set clear limits to avoid negative impacts through higher import dependency, land intensification, and land conversion (Fuchs et al. 2020).

Analyzing this strategy provides insights into the Commission's position and action plan. The concrete development of the bioeconomy depends on how specific EU policies address it (Töller et al. 2021). To our knowledge, only Vogelpohl et al. (2021) look into this issue empirically. Their study indicates that the EC has not yet successfully established the bioeconomy strategy in the proposed holistic and comprehensive way. Instead, the EU’s bioeconomy policy is disjointed and mainly focused on established areas such as bioenergy and plastics (ibid). However, it remains unclear if and how various actors across different economic sectors and policy areas share interests in influencing the bioeconomy on the EU level. In particular, whether conflicts arise between actors in tackling biophysical limits is an open question.

We aim to address the described research gap by analyzing how actors frame and address biophysical limits in conflicts over regulating biomass use in EU policies. To approach this research interest, we base our study on the following research questions: *where are the areas of conflict in which biophysical limits of the bioeconomy are negotiated? How do actors involved in these conflicts frame and address biophysical limits and seek to regulate biomass use accordingly? To what extent can the competing actors assert their positions and strategies in EU policies*?

In an early phase of our empirical research, we identified two conflicts surrounding the bioeconomy strategy in addressing biophysical limits. The first conflict concerns the EC’s proposal to implement cascading use in EU policy (European Commission 2018a; 2022b). Different interpretations of the cascading use exist in the literature, but they all emphasize utilizing biomass multiple times before its energetic use and disposal (Avitabile et al. 2023). If executed effectively, cascading use can reduce the use of primary biomass for energy, mitigate competition on feedstocks and land, and decrease pressure on ecosystems (Bais-Moleman et al. 2018; Haberl and Geissler 2000). But, stricter regulation of biomass markets is at odds with the dominant neoliberal orientation of the bioeconomy project (Vogelpohl 2023; Birch et al. 2010). The second conflict concerns the production of bio-based plastics, which the EU bioeconomy strategy promotes as an opportunity to tackle global plastic pollution (European Commission 2018a). In the discussion about bio-based plastics, the fundamental conflict of the bioeconomy becomes clear. The general narrative describes biomass as renewable by definition. This means that large-scale substitution of fossil fuels is regarded as sustainable without restriction (Boyer et al. 2022). Yet, some actors question this assumption and prioritize a general reduction in plastic production.

To examine the two conflicts, we combine our research framework rooted in critical policy analysis (CPA) (Brand et al. 2022; Buckel et al. 2014; Pichler 2023) with insights on biophysical limits of biomass flows and land use (Haberl 2015; Kalt et al. 2020). We identify and group actor positions and strategies into three competing political bioeconomy projects (Buckel et al. 2014). To shed light on the potential of actors to assert their interests in the two conflicts, we examine which projects can shape if and how EU policy documents address biophysical limits. As an empirical foundation of our research, we qualitatively analyzed interviews, position papers of actors, and EU policy documents (Mayring 2015; Blatter et al. 2018).

By analyzing particular policy conflicts, our study outlines the areas where the biophysical limits of the bioeconomy are currently being negotiated. We summarize similar or competing positions and strategies of various societal and political actors. This abstraction of three competing bioeconomy projects enables a better understanding of contradictions and alliances in shaping the bioeconomy project. As we examine which positions and strategies prevail in specific EU policies, we can draw conclusions about the potentials and barriers under the current political–institutional setting to tackle biophysical limits. The upcoming sections are organized in the following manner. In the next section, we present our analytical framework based on critical policy analysis. We furthermore describe the empirical material and methods we used. In “Results”, we first demonstrate the abstraction of the three competing bioeconomy projects. Following this, we examine how the actors allocated to these projects react to the two conflicts and to what extent they inscribe their positions and strategies in bioeconomy policies. We relate our results to a political–economic and biophysical context in the “Discussion”. Finally, we summarize the primary outcomes in “Conclusion” and suggest potential areas for further research.

## Analytical framework and methods

Critical policy analysis assumes that policies are not the result of rational decision-making and cannot merely be analyzed in terms of their effectiveness for problem-solving. CPA describes policy development as a strategic field of competing actors that seek to assert their interests (Brand et al. 2022; Caterina 2018). Hence, actors’ interests and strategies in shaping a political project become apparent in policy conflicts (Buckel et al. 2014). CPA suggests a broad definition of “actor” to analyze conflicts beyond the political institution, parties, and their personnel. This definition acknowledges that organizations or individuals in the societal sphere, such as non-governmental organizations (NGOs), industry associations, private companies, experts, and researchers, are also interested in or actively participate in policy development (Kannankulam and Georgi 2014; Brand et al. 2022).

For analyzing conflicts over environmental policies, CPA draws on the theory of societal relations to nature (Schneider et al. 2023; Brand et al. 2022). This theory postulates that a capitalist society must reproduce its relations to nature in a way that enables the accumulation of capital. This specific relation causes an ongoing attempt to control and exploit natural resources and sinks without considering their particular biophysical characteristics and limits (Jahn and Wehling 1998; Görg 2003). Accordingly, environmental policies are highly contradictory. Over the last two decades, political discourses have increasingly acknowledged the urgency to tackle the ecological crisis. Nevertheless, policies aim to reconcile addressing this crisis with economic growth. Thus, policies tend to strategically focus on market-based policy solutions and disregard the implementation of preemptive legal measures to uphold biophysical limits (Pichler et al. 2020; Brand 2016). This strategic orientation ignores that economic growth is the key driver for increasing resource use and environmental degradation (Krausmann et al. 2009; Haberl et al. 2020; Hickel 2019). Consequently, the opportunities for a radical reduction in the use of resources are structurally limited (Brand 2016). Nonetheless, individual political institutions, ministries, and personnel can pursue partially conflicting interests and strategies (Schneider et al. 2023). Furthermore, international environmental politics is shaped by particular and often competing national interests (Brand 2010). Social actors can ally with political institutions to assert their interests and strategies. Acknowledging this complexity is essential to analyze the EU bioeconomy, which incorporates various Directorate-Generals (DGs) and Member States, and thus, different perspectives and agendas (Vogelpohl et al. 2021; Töller et al. 2021). As elaborated above, the bioeconomy project also mainly seeks to develop new opportunities for economic growth through a large-scale mobilization of biological raw materials (Eversberg et al. 2023). By understanding policies as a conflicted field, CPA enables us to examine to what extent actors are defending or questioning this growth-driven project and how they can shape the regulation of biomass in EU policies.

As a first step of the CPA, we reviewed quantitative studies on the impacts of a growing bioeconomy on global biomass flows and land use (Erb and Gingrich 2022) to develop a set of deductive codes. We adjusted these codes and created inductive codes during our research, adding more information on actors' positions and strategies (Table 1). We used the predefined coding scheme to screen all 62 position papers of actors on the European Commission’s (EC) recent bioeconomy roadmap initiative (European Commission 2021a). During this screening, we searched for controversial discourses that might indicate the broader conflicts in developing EU bioeconomy policy. To expand the identification of conflicts, we conducted four exploratory interviews (Blatter et al. 2018) (Interview guideline S3) with bioeconomy experts from academia, policy-making, and industry (Table S2).

**Table 1:** Coding scheme for qualitative content analysis

Code group	Research question	Code examples
Biomass feedstocks	With what feedstocks do actors seek to substitute fossil raw materials?	*BF_biomass feedstocks_secondary biomass flows_agricultural residues*
Biophysical limits	What biophysical limits do actors consider in the context of bioeconomy, and how?	*BL_biophysical limits_land use competition*
Strategies for biophysical limits	How do actors propose to address biophysical limits?	*STO_strategies for biophysical limits_sustainability criteria and standards_for primary production*
Positions on cascading use	How do actors react to the proposal to implement cascading use in policy?	*CF_position on conflict_cascading use_against imposing a legislative implementation*
Positions on bio-based plastics	How do actors position themselves to increase, decrease or limit bio-based plastics production in the EU bioeconomy?	*CF_position on conflict_bio-based plastics_no support_single-use plastics*
Regulation strategy	How do actors propose to regulate biomass use in the context of biophysical limits?	*RS_regulation strategy_market-based regulation_certification and labeling*

In the next step, we analyzed 23 of the 62 position papers (PP#) (Table S1) on the roadmap initiative in more detail to refine our coding scheme. We examined the EU bioeconomy strategy to understand how the European Commission addresses the two conflicts. We used the coding scheme to create a semi-structured guideline for the expert interviews (see interview guideline S3). We conducted eight semi-structured interviews (Galletta 2013) with actors from biomaterials and bioenergy industries, agriculture, forestry, EU institutions, academia, and NGOs. We anonymized all interviews and only refer to the interviewees with a general description of their actor group and a codename (INT#) (Table S2). We transcribed the interviews and coded relevant paraphrases according to the explicit statements of interviewees and our interpretations of implicit meaning (Galletta 2013; Kaiser 2014). Additionally, we analyzed nine position papers (Table S1) on the Commission's initiative for an EU policy framework on bio-based, biodegradable, and compostable plastics (European Commission 2022a).

We then analyzed the individual statements to identify the actors’ central positions and strategies. When referring to positions, we describe how actors generally describe the biophysical limits of the bioeconomy and react to the identified conflicts (Buckel et al. 2014). We examine the overarching strategic orientation to address biophysical limits, such as relying on growth or reducing resources. Additionally, we analyze specific proposals for regulating biomass use (Sander 2016). Based on these positions and strategies, we categorized the different actors into three competing political projects that aim to shape the bioeconomy’s development (Buckel et al. 2014). These projects give us a heuristic of potential alliances of actors and the power relations in shaping policies (Brand et al. 2022; Pichler and Ingalls 2021). It is important to note that political projects are abstractions and do not exist as concrete entities. Actors may not necessarily be aware of how they are connected or separated from one another. They may also have contrary particular interests, sometimes overlapping with other projects. What unites them is a shared strategic orientation (Buckel et al. 2014). In our research process abstracting the bioeconomy projects from the empirical material followed the identification of conflicts. However, in the results section of this article, we first describe the abstraction of bioeconomy projects and then show the extent to which the three different projects appear in the conflicts and are inscribed in the particular policies.

Ultimately, we used the final set of codes to analyze policy documents revealing if and how competing bioeconomy projects could inscribe their positions and strategies on biophysical limits in the two conflicts. For the conflict on cascading use, we analyzed the provisional agreement on the second revision of the Renewable Energy Directive (RED III) (Council of the European Union 2023). For the conflict on bio-based plastics, we analyzed the Single-Use Plastics Directive (SUP) (European Parliament and Council of the European Union 2019) and the EU policy framework on bio-based, biodegradable, and compostable plastics (BBCPs) (European Commission 2022a).

## Results: negotiating biophysical limits in the bioeconomy

### Three competing projects in negotiating biophysical limits of the bioeconomy

Table 2 shows the three bioeconomy projects that diverge in how they address biophysical limits. We abstracted actors from agriculture, the bioenergy industry, the biomaterials industry, and forestry into a *growth-oriented bioeconomy project.* This project pursues a significant increase in primary and secondary biomass supply to substitute fossil raw materials. Thus, “it is important that the EU ensure it fully utilizes its biogenic resources and avoids placing new restrictions limiting its sustainable growth.” (PP 2, bioenergy association) Yet, actors do not specify how much biomass flows can be mobilized in the future. They merely promote establishing standards and criteria for producing feedstocks of bioeconomy products to change consumer behavior (INT 5 and 6; PP 2, 5, 8, 13, 16, 18, 24, and 32).

**Table 2:** Three competing bioeconomy projects in negotiating biophysical limits

	Growth-oriented bioeconomy project	Circular bioeconomy project		Sufficiency-oriented bioeconomy project
Actor groups	Agricultural and forestry associations; bioenergy industry; biomaterials industry	Academia; circular biomaterials industry; waste management industry; public organizations		Academia; NGOs
Positions on biophysical limits of the bioeconomy	Policy should not restrict biomass use	Primary biomass and land are limited; growing bioeconomy can induce land use competition		
Main strategies to address biophysical limits	Preventing restrictions on biomass use; additionally mobilizing secondary biomass flows	Mobilizing secondary flows; fostering circularity; reduction of feed production		Absolute reduction of resource use; sufficiency-oriented provisioning system
Position on cascading use	Hampers growth of the bioeconomy; reduces efficiency; disregards regional circumstances	Increases resource efficiency and material use of biomass; reduces competition on biomass and land; reduces pressure on carbon stocks and biodiversity		
Strategy for regulating cascading use	Market dynamics and prices	(Re-)allocating subsidies		Ending subsidies for bioenergy from primary biomass; binding caps for bioenergy from primary biomass; binding rules on EU level
Positions on bio-based plastics	Increasing the production of all bio-based plastics; current EU policy is a threat to expanding the production of bio-based plastics	Limited support for bio-based plastics from primary biomass and single-use plastics;		Reducing single-use products; no support for plastics from primary biomass; prioritizing reuse and longevity;
		Fostering recyclability; supporting plastics from secondary biomass		
Strategy for regulating bio-based plastics	Standardization, certification, and labeling; binding targets for increasing the use; minimum content of biomass in products	Guidelines for waste collection schemes; binding recycling targets; standards for bio-waste quality		Binding targets for waste prevention and reuse; EU-wide reuse system; binding sustainability requirements

We abstracted academic institutions, industries producing circular materials, the waste management industry, an EU counselor, and public organizations into a *circular bioeconomy project*. This project emphasizes a limit in mobilizing primary biomass for a growing bioeconomy due to increased competition on land and feedstocks and pressure on ecosystems (INT 2 and 10; PP 7 and 20). Instead, the EU can satisfy its future demand with wastes, residues, and by-products (INT 2 and 10; PP 1, 4, 6, 7, 10, 17, 20, and 22). Furthermore, actors from waste management and one EU counselor suggest using less area for feed production to increase the availability of land for the bioeconomy while ensuring food security (INT 2 and 10; PP 7 and 10).

We abstracted individual bioeconomy scholars, one actor working for the EC, and environmental NGOs into a *sufficiency-oriented bioeconomy project*. This project also acknowledges that a growing bioeconomy can aggravate land use competition and pressure on ecosystems (INT 1, 3, 8, 11, and 12; PP 12, 25, and 37). Nevertheless, it questions whether increasing resource efficiency and circularity alone can keep the bioeconomy within its limits (INT 3, 8, and 12). Thus, significant shifts in provisioning systems for mobility, energy, and food are necessary to reduce overall resource use (INT 1, 3, 8, 11, and 12; PP 12 and 33).

### Negotiating cascading use in the EU bioeconomy

The bioeconomy strategy promotes cascading use to foster resource efficiency and increase the supply of wastes, residues, and by-products. It suggests implementing cascading use through effective regulation and responsible practices to mitigate the environmental impacts of bioenergy (European Commission 2018a; 2022b). Thereby, cascading use should adhere to the “following order of priorities: wood-based products; extending their service life; re-use; recycling; bioenergy; disposal.” (European Commission 2022b) While this definition only focuses on woody biomass, the bioeconomy progress report seeks to extend the cascading use to all types of biomass (ibid.). Beyond this technical dimension, cascading use is strategically vital for the European Commission to develop a sustainable and holistic bioeconomy (INT 1, 3, 4, and 10). Cascading use is an approach to link material and energy industries by setting rules on which feedstocks can be used for energy and what is prioritized for material utilization (INT 10). It aligns with the Commission’s objective that “bioeconomy policies take a cross-sectoral perspective to improve policy coherence and identify and resolve trade-offs, for example, on land and biomass demands.” (European Commission 2022b) Yet, actors within and outside the EC contest the definition and policy integration of cascading use (INT 3, 5, and 10).

#### Actor’s positions and strategies on implementing cascading use in EU policy

##### The growth-oriented bioeconomy project

Actors from the bioenergy industry, forestry, and agriculture oppose implementing cascading use in EU policies. They posit that this restricts the substitution of fossil fuels with biomass, hampering growth, innovation, and jobs (INT 4, 5, and 9; PP 5, 9, 11, and 15):

The EU is currently discussing the possibility of regulating biomass markets by enforcing a cascading use of biomass, but compulsory rules that limit market access or impose a cap on the use of biomass in certain industry sectors risk reducing the competitiveness of the whole sector and might well hinder technological advances. (PP 9, forestry association)

Consequently, implementing the cascading use “would ultimately hamper the flexibility our industry needs to thrive and contribute to the EU climate targets.” (PP 11, biochemical company) Instead, these actors endorse a market-based approach of cascading use, acknowledging diverging economic and territorial circumstances in Europe (INT 5 and 9; PP 2, 8, and 11). Accordingly, market prices would incentivize primary producers not to use or sell higher-value biomass for energy production:

It is not that forest owners use the best quality wood for energy because energy is usually the lowest-paid category. [..] Of course, we are trying to always get the best value out of our forests. (INT 9, forestry association)

##### The circular and sufficiency-oriented bioeconomy projects

The circular and sufficiency bioeconomy projects share the position that cascading use increases resource efficiency and biomass utilization for materials. Furthermore, it reduces competition on biomass and land and minimizes pressure on ecosystems and biodiversity (INT 2, 3, 11, and 12; PP 1, 6, 10, 12, and 17). Furthermore, actors of the EC, waste management, and NGOs promote the cascading use to limit bioenergy. They stress that expanding bioenergy production decreases carbon sinks and biodiversity in forests (INT 2, 3, 11, and 12; PP 12):

And when trees are re-planted it takes 50 to 100 years before the CO2 is again absorbed. […] when you replant forests, you get cultivated forests, a plantation with trees without any biodiversity. (INT 11, environmental NGO)

As a regulation strategy, the EC official and NGOs suggest removing subsidies for processes not adhering to cascading use (INT 3, 11, and 12). Additionally, NGOs propose introducing binding caps and ending subsidies for bioenergy from primary biomass (INT 11 and 12). Despite that, most actors do not specify the scale of regulation. One EU counselor states that the interpretation "should be given to the local level and the regional level" (INT 10) due to geographical, climatic, and socio-economic differences among EU regions. Only one NGO actor calls for defining and implementing cascading use on the EU level (INT 11). In the following section, we demonstrate how these three bioeconomy projects have incorporated their positions and strategies on biophysical limits and cascading use into RED III.

#### Implementing cascading use in the second revision of the renewable energy directive

In July 2021, the EC proposed to revise the renewable energy directive for the second time. The main endeavor was to increase the initial renewable energy target from 32 to 42.5% by 2030. In line with the position of the growth-oriented bioeconomy project, RED III promotes bioenergy as a vehicle to reach this target (European Commission 2021b). NGOs criticize that a higher target without stringent limits on bioenergy might incentivize the increasing use of wood (INT 11 and 12; PP 12). Still, RED III incorporates the diagnosis of the circular and sufficiency-oriented project that using crops for bioenergy competes with food production and biomaterials and can lead to indirect land use change. It acknowledges that harvesting woody biomass is limited due to protecting forests and carbon sinks. Thus, RED III determines old-growth forests as no-go areas from which biomass must not be harvested for bioenergy. Yet, every country can define what qualifies as old-growth forests (Council of the European Union 2023). An NGO employee doubts that this amendment can limit woody biomass harvesting (INT 12).

Furthermore, RED III proposes a compromise between the competing bioeconomy projects by regulating cascading use through financial support schemes. Member States can deviate from cascading use if securing energy supply is required or if there are no options for material utilization of feedstocks in the particular region (Council of the European Union 2023). Moreover, RED III does not incorporate the European Parliament’s (EP) initial proposal to remove subsidies and cap harvesting primary woody biomass for bioenergy (INT 11 and 12). An NGO employee considers the EP’s proposal “a robust implementation of the cascading principle […] as like you only keep subsidies for residues of wood processing” (INT 12). Instead, RED III replaces the notion of primary woody biomass with industrial-grade round wood that should be excluded from receiving bioenergy subsidies. Again, Member States can define industrial-grade round wood (Council of the European Union 2023). Furthermore, “Member States should grant no direct financial support to the production of energy from saw logs, veneer logs […] stumps and roots.” (Council of the European Union 2023) Nonetheless, granting tax benefits for producing energy from these materials and industrial-grade roundwood does not count as direct financial support and is thus still allowed (ibid.). In October 2023, the EU officially approved RED III, requiring Member States to adjust their national legislation on renewable energy within 18 months. Actors of the circular and sufficiency project doubt the effectiveness of the current take on cascading use. Forest-rich Finland, Sweden, and some Eastern European countries highly promote biomass as renewable energy and will probably use the freedom given to them (INT 3, 10, 11, and 12).

### Negotiating the production of bio-based plastics in the EU bioeconomy

The bioeconomy strategy aims at “solving the problem of plastic litter in seas and oceans via the support to research and innovation for the development of alternatives to fossil-based plastics.” (European Commission 2018a) It suggests developing standards and labeling on the biodegradability of bio-based plastics to ensure their sustainability and boost their market uptake. Furthermore, the strategy promotes using wastes and residues to produce bio-based plastics. However, it does not explicitly describe to what extent bio-based plastics can be made from primary biomass within biophysical limits (European Commission 2018a). Because of current EU policies promoting the reduction and circularity of plastic products, we identified a conflict between different actors. We analyze the positions and strategies of these actors in the following.

#### Actors’ positions and strategies on regulating bio-based plastics in EU policy

##### The growth-oriented bioeconomy project

Actors from the biomaterials industry, forestry, and agriculture support the production of single-use and circular bio-based plastics. Their fundamental premise is that plastics made from biomass are CO_2_-neutral, thus contributing to climate change mitigation (PP 11, 18, 21, 24, and 32). They emphasize the biodegradability of these plastics, i.e., closing carbon cycles. This assumption implies that increasing biomass use for plastics bears no threats to ecosystems and carbon stocks (INT 7; PP 14 and 21). Most of these actors do not address the biophysical limits of bio-based plastics. Two industry actors admit that using primary biomass can increase land use competition with food and might endanger biodiversity-rich ecosystems (INT 7; PP 30). Overall, this project regards the current EU policy as a threat to the sector (INT 5, 6, and 7). To counteract this tendency, industry actors promote establishing sustainability criteria and standards to stimulate market uptake of bio-based plastic (INT 6 and 7; PP 16, 18, 21, 24, and 32). Furthermore, they urge the EU to introduce binding targets for increasing bio-based plastics use and establishing minimum biomass content in products (INT 6; PP 24).

##### The circular bioeconomy project

The position on bio-based plastics by actors of the circular project is ambiguous. The EU counselor explicitly supports single-use bio-based plastics:

My general opinion is where we can help to use bio-based carbon instead of fossil-based carbon we should help it and we should try to do our best. And this is the case also in the single-use plastics. (INT 10)

One research institution emphasizes biodegradability to reduce the environmental impact and increase the circularity of bio-based plastics (PP 20). One waste management actor argues that biodegradability is a technically complex concept and depends on the specific environments where products end up. Thus, biodegradability is not guaranteed (PP 19). Furthermore, one bio-based plastics producer elaborates that single-use plastics uphold a linear model, leading to increasing demand for primary biomass. Thus,

incineration, biodegradability, and compostability as end-of-life scenarios for plastic materials should only be applied in case reuse or recycling are not feasible, as the embedded carbon is irretrievable lost from the material circle afterwards. (PP 36)

One waste management actor acknowledges that substantially increasing the global demand for bio-based plastics from primary biomass can aggravate land use competition:

[…] by the end of this decade, plastics production will go to 500 or 600 million tons. As of the scale compared, bioplastics use is tiny. But ten million tons sort of becomes an amount, which you will find for sure in certain places, that begins to compete with food. (INT 2)

Accordingly, waste management and industry actors promote producing recyclable plastics and plastics from secondary biomass to reduce the risk of land use competition (INT 2; PP 9 and 36). These actors encourage recycling targets, organic waste collection schemes, and waste quality standards as policy measures to increase the production of such products (INT 2; PP 7, 17, 22 and 36).

##### The sufficiency-oriented bioeconomy project

NGOs oppose the production of single-use plastics made from biomass due to similar concerns expressed by the circular bioeconomy project (INT 12; PP 25, 34, and 37). Furthermore, they do not support the production of bio-based plastics from primary biomass, which can increase

pressures on land, particularly where their production is supported by intensive and fossil-fueled agriculture, and may not by default perform any better than their fossil-based counterpart from an environmental and circularity perspective. (PP 33)

Thus, they suggest an absolute reduction of all types of plastics, especially single-use plastics, and foster the reuse and recycling of plastics (PP 25, 33, and 34). NGOs further argue that voluntary labeling of bio-based plastics cannot ensure sustainability and biodegradability, leading to greenwashing. Therefore, they encourage the development of legally binding standards for bio-based plastics with low environmental impacts that do not cause indirect land-use change (PP 25 and 37).

In the following section, we analyze to what extent the competing projects were able to inscribe their positions and strategies into bio-based plastics policy.

#### Regulating bio-based plastics in the single-use plastics directive

The Single-Use Plastics Directive entered into force on July 2nd, 2019. SUP seeks to counteract the waste and pollution induced by the overproduction of single-use plastics. For these plastics, “related production and consumption patterns have become increasingly inefficient and linear” (European Parliament and Council of the European Union 2019). In line with the position of the sufficiency project, SUP suggests reducing the production and consumption of single-use plastics, including bio-based ones. Furthermore, SUP incorporates the demands of circular and sufficiency-oriented bioeconomy projects to emphasize the production of recyclable and reusable bio-based plastics. The growth-oriented bioeconomy project could not assert its strategy of producing all types of bio-based plastics. Thus, a manufacturer complains that SUP

restricts policy measures at member states to contribute to the global challenge of plastic-free oceans based on innovative bio-based products, and it restricts further innovations in this field. (PP 21)

SUP requires Member States to reduce the consumption of single-use plastics with no available alternatives (hygiene articles, tobacco products, beverage cups, food containers, packets, wrappers, etc.) by 2026. It suggests national reduction targets, developing reusable plastics, or changes in taxation for single-use plastics to achieve this reduction. In addition, SUP proposes a market-based approach in the form of labels for products intended for single use. This form of encouragement (instead of a regulation) of consumer behavior is in line with the strategy of the growth-oriented project. Furthermore, SUP suggests developing a standard for the marine biodegradability of single-use plastics. Consistent with the position of the sufficiency project, SUP requires Member States to prohibit putting single-use plastic products (cotton bud sticks, cutlery, plates, straws, and beverage stirrers) with suited and sustainable alternatives on the market (European Parliament and Council of the European Union 2019).

Overall, SUP focuses more on addressing the global issue of environmental pollution and counteracting the linearity of production systems through regulation. However, it does not explicitly consider the central limit when switching to bio-based—the potential of land to provide sufficient feedstocks. Thus, to gain more insights into this issue, we analyze the EU policy framework on BBCPs in the following.

#### Regulating bio-based plastics in the EU policy framework on bio-based, biodegradable, and compostable plastics

The EU policy framework on BBCPs was published on November 30th, 2022, to guide future EU policy-making on bio-based plastics (European Commission 2022a). The framework accentuates that producing bio-based plastics from primary biomass

can lead to direct or indirect land-use change, resulting in biodiversity loss, ecosystem degradation, deforestation and water scarcity, as well as competition with crops intended for human consumption. (European Commission 2022a)

It points out that substituting single-use plastics can significantly increase the use of biomass as they have a short lifetime and need continuous material inputs. Promoting bio-based plastics should not distract from reducing single-use plastics and resource use in general. Furthermore, the framework prioritizes recycling, reusing, and utilizing secondary feedstocks. Accordingly, it refers to cascading use to support bio-based plastics production. Hence, “biomass should be preferably used to produce materials, including plastics, and only in subsidiary order, as a source of bioenergy.” (ibid., p. 5) This strategic orientation resembles the positions of the sufficiency-oriented and circular bioeconomy projects.

For applications where reduction, recycling, and reuse are not feasible, the framework takes up the growth-oriented project’s strategy to develop standards and labeling for biodegradability. However, the framework concedes that developing standards on marine biodegradability might not be feasible as “biodegradation at the bottom of the ocean is unlikely due to the specificities of the marine environment.” (ibid., p. 10) Thus, it underscores the need to develop standards for certifying and labeling products exclusively for proven biodegradability in specific environments. Furthermore, the framework promotes single-use plastics if produced from organic wastes and by-products. If producers use primary biomass, the framework suggests developing criteria that prevent land use change (ibid.).

## Discussion

We identified conflicts over addressing the biophysical limits of the EU bioeconomy. Analyzing these conflicts, we could abstract actors’ positions and strategies into three political projects. The growth-oriented bioeconomy project frames limiting biomass use as a hurdle for economic growth and competitiveness. The positions and strategy of this project are based on the assumption that biomass is a renewable and, thus, sustainable and unlimited resource. If trade-offs occur, this project considers them a problem of the future or solvable through technical innovation and improved management (Boyer et al. 2022; Goven and Pavone 2014). The circular bioeconomy project recognizes a limit in mobilizing primary biomass due to increasing competition on land and feedstocks. It suggests mobilizing secondary biomass while utilizing less land for feed production to increase the bioeconomy’s supply. We found that only the sufficiency-oriented bioeconomy project critiques the dominant focus on substitution and growth, thus calling for reducing resource use through substantial changes in consumption patterns and provisioning systems. Our results indicate that actors who acknowledge biophysical limits do not necessarily translate this diagnosis into measures reducing resource use. For instance, the circular bioeconomy project stresses the potential of secondary biomass and land use shifts to increase biomass availability for material use and, to some extent, for energy. This does not preclude that this project focuses on economic growth and substitution as long as sufficient land, waste, by-products, and residues are available. For instance, promoting cascading use to increase the availability of residues and by-products draws on the assumption that (socio-)technical innovation will solve the problems arising from biophysical limits (Boyer et al. 2022). This strategy does not necessarily consider a potential need to decrease the demand for resources and energy. On the contrary, applying the cascading use in this way can risk overusing residues from agriculture and forestry, leading to carbon and biodiversity losses and degradations of soils (Blanco-Canqui and Lal 2009; Lattimore et al. 2009). Therefore, policy development needs to supplement cascading use with an absolute limit on using biomass for energy to protect ecosystems and forests (Searchinger et al. 2018; Erb et al. 2022). However, REDIII gives Member States significant leeway for implementing the cascading use. In the following, we discuss potential explanations for the outcome of REDIII and how this differs from the bio-based plastics policy.

From the CPA perspective, our analysis indicates that the bioeconomy is still geared toward economic growth and competitiveness. This strategic orientation manifests itself differently across policy fields, and there is some potential for alternative strategies. We found that the sufficiency-oriented and circular bioeconomy projects could discursively inscribe their diagnosis of biophysical limits in both policy conflicts. These projects could shape how the policies regulate biomass use only to a limited degree. The bio-based plastics policy seeks to partially limit the production of bio-based plastics, primarily if they are intended for single-use applications. SUP puts restrictive measures in place for single-use plastics. The EU policy framework on BBCPs additionally outlines a policy strategy prioritizing the reduction of resource use together with the reuse and recycling of plastics. In contrast, regulating cascading use in RED III denotes a compromise with the positions and strategies of the growth-oriented bioeconomy project. It is doubtful that this compromise will limit energy production from primary biomass (Stubenrauch and Garske 2023).

Bio-based plastics are a considerably new sector shaped by the path dependencies established through regulating fossil plastics (Vogelpohl et al. 2021). European policies (European Commission 2018b; European Parliament and Council of the European Union 2015) on plastics are geared towards tackling plastic pollution by reducing plastics and increasing recycling for at least a decade (Calabrò and Grosso 2018). Within the Commission, the Directorate-General for Environment (DG ENVI) mainly pushed this institutional development (Vogelpohl et al. 2021). DG ENVI prioritizes mitigating the environmental impact of plastics over economic growth (European Commission 2018b). Furthermore, this DG also led the development of SUP and the EU policy framework on BBCPs, which can explain current policy orientation. Vogelpohl et al. (2021) note that these developments are also due to a lack of a broad alliance of actors who support developing the bio-based plastic sector. As our analysis starts from the most recent discussions on the bioeconomy strategy, we can broaden this perspective. Accordingly, our results indicate a growing support of forestry and agricultural actors for bio-based plastics as they see opportunities for new sales markets for biomass. In addition, associations such as the EU Bioeconomy Alliance have emerged in recent years to pool the interests of various sectors such as bioenergy, biochemistry, forestry, agriculture, and bio-based plastics. This alliance also explicitly advocates for fostering the production and use of bio-based plastics in the EU (European Bioeconomy Alliance 2023). The current EU policy also supports bio-based plastics despite focusing on reduction and circularity. Both SUP and the EU policy framework on BBCPs propose measures to promote research and labeling of biodegradability, which is seen as an asset of some bio-based plastics compared to conventional plastics. Furthermore, neither policy document intends to restrict the production of recyclable and long-lasting products. This policy context and the growing alliance of bio-based sectors might still lead to a significant growth in the demand for bio-based plastics. For instance, Plastics Europe projects that the use of bio-based plastics in the EU will rise from 1 million tons in 2021 to 11 million tons in 2050. For this calculation, the industry association assumed that in 2050, 50% of bio-based plastics will still be produced from primary biomass (Plastics Europe 2023). This potentially growing use of bio-based plastics can increase land use (Brizga et al. 2020; Escobar et al. 2018; Escobar and Britz 2021) and aggravate the competition with food production and protection of ecosystems (Haberl 2015).

Our analysis of RED III shows that actors of the growth-oriented bioeconomy project could hinder a rigid interpretation of cascading use. An alliance of bioenergy producers, agriculture, forestry sectors, and forest-rich Member States has promoted bioenergy as a renewable energy and growth sector for decades and created path dependencies for the EU to increase its use (Banja et al. 2019; Stupak et al. 2007; Vogelpohl et al. 2021). The use of biomass for energy in the EU increased from 154,9 million tons in 2009 to 207,3 million tons of biomass in 2017 (Gurría Albusac et al. 2017). In comparison, the total production of bio-based plastics in the EU amounted to 1 million tons in 2021 (Plastics Europe 2023). Furthermore, 22% of all biomass flows in the EU are used to produce energy. For woody biomass, as much as 49% is used for energy (European Commission 2022b). The vast difference in mass alone indicates that bioenergy is of much greater economic and political interest than bio-based plastic. Our results illustrate that the agricultural and forestry sectors consider bioenergy a significant sales market. Therefore, these sectors have a material interest that the EU does not restrict biomass production and strives for market-oriented regulation. Furthermore, bioenergy fulfills the EU’s strategic role in achieving its renewable energy targets (European Commission 2021b). Russia’s attack on Ukraine and the following energy crises strengthened the ambition of the EU to increase energy independence (European Commission 2023a). In the latest EU bioenergy sustainability report, biomass accounted for 59% of renewable energy consumption in 2021 (European Commission 2023b). Finally, reducing bioenergy production in the EU makes it more difficult for Member States to reach their renewable energy targets (Proskurina et al. 2016). Due to this interest and also to support their industries, Member States with extensive forest areas and industries, including Finland, Sweden, and some Eastern European and Baltic countries, were actively working to impede the adoption of stricter cascading regulations and limitations on using woody biomass for energy. For instance, these Member States submitted a letter to the Council and Commission to influence the RED III negotiations. In the letter, they expressed their disagreement with the Commission's proposal for stricter rules on bioenergy because “the proposed delegated powers for the Commission on the cascading principle will further increase the uncertainty of the bioenergy regulatory framework.”(Farmanbar et al. 2021)

## Conclusion

Through critical policy analysis, we explored how competing political bioeconomy projects in the EU address biophysical limits and to what extent they can assert their positions and strategies in two policy conflicts. We illustrate that the analyzed policies at least rhetorically take up concerns that an ever-growing bioeconomy exceeds the reproductive capacity of land and ecosystems. Looking at the case of bio-based plastics, in particular, indicates that biophysical limits have become increasingly important and that measures to reduce resource use are finding their way into EU policy. Our findings further indicate an overlap of the positions of NGOs with those of academia, public institutions, and parts of the biomaterials industry and waste management. This can be the basis of future alliances pushing the development of a sustainable bioeconomy within biophysical limits. However, in alliance with forest-rich Member States, actors from agriculture, forestry, and the bioenergy industry could dilute putting clear limits on bioenergy in REDIII, indicating a hurdle for addressing biophysical limits.

By abstracting three competing bioeconomy projects, we comprehensively analyze diverging positions and strategies on biophysical limits. Still, this remains a rough abstraction that does not rule out variability and potential overlaps between the projects. Furthermore, the three projects cannot portray the positions and strategies of all political and societal actors potentially involved in the bioeconomy (e.g., Brand et al. 2022). The selection of empirical material to develop this abstraction started with the position papers on the Commission’s bioeconomy roadmap. We expanded the empirical material by interviewing designated bioeconomy experts representing various societal and political actors. CPA enables a broad range of interests and strategies that enter the formal policy process and are, therefore, visible. However, with this approach, it is challenging to cover actors who did not actively participate in the feedback process but are interested in the bioeconomy or are affected by it. We furthermore could identify to what extent the different positions and strategies overlap with the analyzed policy documents. Within the scope of this research and the selection of empirical material, we did not investigate the concrete institutional settings and power dynamics filtering how projects asserted their positions in more depth. To analyze the power relations underlying the development of bioeconomy policy, further research can draw on the concepts of strategic selectivity (Jessop 1999) and power resources (Kannankulam and Georgi 2014). Combining these two concepts makes it possible to analyze actors’ interests, strategies, and structural conditions more precisely and systematically.

For further policy development, we can conclude that limiting and reducing resource use in addition to (socio-)technological innovations is necessary. Furthermore, we note that the criticism of the bioeconomy is not a plea for fossil fuels. The transformation towards a post-fossil society is inevitable in tackling the ecological crisis, and the bioeconomy can play a significant role. Thereby, it is essential to link the reduction of resource use with socioeconomic processes and people's everyday practices to make a sustainable bioeconomy just and fair (Ramcilovic-Suominen et al. 2022a, b). Accordingly, the EU’s Joint Research Centre (JRC) has already outlined the future policy development for a sustainable bioeconomy in a recent report. This report highlights that EU policy must adopt a global perspective prioritizing human rights and fair working conditions. Thus, the JRC emphasizes the need to avoid perpetuating global injustices and ensure that bioeconomy innovations benefit everyone. As transforming labor is also a crucial part of a sustainable and equitable bioeconomy, the JRC suggests taxing resource use instead of work as a first step. Finally, bioeconomy policy must develop beyond its sectoral fragmentation and promote inclusivity and transdisciplinarity. Thereby, it is crucial to remain open to oppositional critique and strengthen democratic processes that include citizens in decision-making (Giuntoli et al. 2023). From the CPA's perspective, future research can take a more explicit look at the extent to which actors at the EU and national level link this vision of a just and sustainable bioeconomy to concrete strategies.

## Supplementary Information

Below is the link to the electronic supplementary material.

Supplementary file1 (DOCX 29 KB)

## Data Availability

The data that support the findings of this study are available from the corresponding author upon reasonable request.
