# Supplementary material for: Negotiating biophysical limits in the European Union’s bioeconomy: a critical analysis of two conflicts over regulating biomass use in EU policy
Source: Sustain Sci. 2024 Aug 14;19(6):1935–48. doi: 10.1007/s11625-024-01543-0 (PMC11543732; doi:10.1007/s11625-024-01543-0)
Supplement: Supplementary file 1 — Supplementary file1 (DOCX 29 KB) [file 11625_2024_1543_MOESM1_ESM.docx]

**Supplementary information**

**Tables**

**Table S1:** Position papers on the European Commission’s initiative European bioeconomy policy: stocktaking and future developments

| **Actors** | **Code** | **Scale** |
| --- | --- | --- |
| Public organization | PP 1 | EU |
| Industry association | PP 2 | EU |
| Agricultural association | PP 3 | National |
| Public organization | PP 4 | National |
| Forestry association | PP 5 | EU |
| Consultancy | PP 6 | National |
| Multi-actor coalition | PP 7 | EU |
| Agricultural association | PP 8 | EU |
| Forestry association | PP 9 | EU |
| Industry association | PP 10 | National |
| Private company | PP 11 | National |
| Non-governmental organization | PP 12 | National |
| Industry association | PP 13 | EU |
| Multi-actor coalition | PP 14 | EU |
| Industry association | PP 15 | EU |
| Multi-actor coalition | PP 16 | EU |
| Multi-actor coalition | PP 17 | EU |
| Industry association | PP 18 | EU |
| Industry association | PP 19 | National |
| Research organization | PP 20 | EU |
| Private company | PP 21 | National |
| Industry association | PP 22 | EU |
| Industry association | PP 23 | EU |
| Private company | PP 24 | National |
| Non-governmental organization | PP 25 | National |
| Industry association | PP 28 | EU |
| Private company | PP 30 | National |
| Non-governmental organization | PP 33 | EU |
| Non-governmental organization | PP 34 | EU |
| Private company | PP 36 | National |
| Non-governmental organization | PP 37 | National |

**Table S2.** Expert interviews conducted between January and July 2023

| **Experts** | **Thematic focus** | **Code** | **Scale** |
| --- | --- | --- | --- |
| Bioeconomy scholar 1 | Exploratory/bioeconomy in general | INT 1 | National |
| Waste management professional | Exploratory/bioeconomy in general | INT 2 | EU |
| European Commission | 1. Interview: Exploratory/bioeconomy in general; 2. Interview: Focus on cascading use and bio-based plastics | INT 3 | EU |
| Biotechnology association | Exploratory/bioeconomy in general | INT 4 | EU |
| Agricultural association | Agriculture; cascading-use; bio-based plastics | INT 5 | EU |
| Biochemical industry professional | Cascading use; bio-based plastics | INT 6 | National |
| Bio-based plastics professional | Bio-based plastics | INT 7 | National |
| Bioeconomy scholar 2 | Bioeconomy in general; cascading use | INT 8 | National |
| Forestry association | Forestry; cascading use | INT 9 | EU |
| Council of the European Union | Bioeconomy in general; cascading use | INT 10 | EU |
| Environmental NGO | Cascading use | INT 11 | National |
| Environmental NGO | Bioeconomy in general; Cascading use | INT 12 | EU |

**Interview guideline S3**

**Short description:** In my current research, I am working on actors, strategies, and policy processes in developing the bioeconomy in the European Union. My focus is on how different groups understand and address the bioeconomy in the context of opportunities and limitations for sustainability.

**Consent:**

I would record the interview for my analysis but only use the statements anonymously, save the recording locally on my computer, and delete the audio files after transcribing them. Therefore, before we begin, I would like to ask if you agree that I record the interview and use your statements for my research.

***Exploratory Interviews***

Introductory questions:

- What does bioeconomy mean to you? What are the most important aspects?
- What is your role in the bioeconomy? How long have you been involved with the bioeconomy?

**Topics**

**1. An understanding of bioeconomy**

Introductory question:

- In your view, what is the central goal of the bioeconomy?

Exploratory question:

- What is the contribution of the bioeconomy to sustainability and the fight against climate change and biodiversity loss?

**2. Importance and role of the bioeconomy strategy**.

Introductory question:

- Where do you see the main contributions of the EU Bioeconomy Strategy to the development of the bioeconomy in Europe?

Exploratory questions:

- Why do you think engaging in the EU Bioeconomy Strategy is essential?
- For which sectors and industries do you think the bioeconomy is particularly important?
- How coherent is the EU bioeconomy strategy so far? Do you see any conflicting goals?
- What are the social and economic impacts and benefits of the bioeconomy?

**3. Actors**

Introductory question:

- In your experience, who have been the actors mainly involved in driving the bioeconomy strategy at the EU level?

Exploratory questions:

- How did these actors drive the bioeconomy strategy?
- Which actors do you think were not involved in developing the bioeconomy strategy and why?

4**. Biomass and land use**

Introductory question:

- To what extent do you think an increase in the supply of biomass in Europe is necessary for the bioeconomy to develop?

Exploratory questions:

- To what extent is it possible to increase the biomass supply in the EU?
- What problems or trade-offs might arise if we increase the supply of biomass? If there are trade-offs, how would they try to resolve them?

***Thematic interviews: cascading use***

Introductory question**:**

- What is your connection to bioeconomy and bioenergy? How were you introduced to this topic, and when did it happen?

**Topics:**

**1. EU Bioeconomy Strategy**

Introductory question:

- In your opinion, what should be the primary objectives of promoting the bioeconomy in Europe?

Exploratory questions:

- How is the sustainability of biomass addressed in the EU bioeconomy strategy?
- To what degree can European agriculture and forestry satisfy the biomass demand for energy and materials? Where do you see trade-offs that might occur when we substantially increase the demand for biomass for biomaterials and energy in the EU?
- What changes in biomass production and consumption have to happen to transition towards a sustainable and just bioeconomy? What changes in land use?

**2. Sustainability of bioenergy**

Introductory question:

- To what extent can bioenergy production help mitigate climate change and promote sustainable resource use in the European Union?

Exploratory questions:

- What are the challenges and trade-offs involved in increasing bioenergy production?
- What regulatory framework is necessary to ensure that bioenergy production in the EU remains within ecological limits?
- The recent progress report on bioeconomy highlighted and promoted the policy implication of cascading use to optimize biomass utilization in a bioeconomy and to limit the use of biomass for bioenergy. What are your thoughts on that? To what extent can this be a solution?

**3. Renewable Energy Directive**

Introductory question:

- What are your thoughts on the recent RED III agreements related to bioenergy production?

Exploratory questions

- Can we rely solely on national supporting schemes for cascading use to ensure sustainable bioenergy production? Or should we explore additional solutions?
- How is the bioeconomy strategy of the EU related to the development of this directive?
- What actors and interest coalitions drove the RED III negotiations, and how?

**4. Outlook**

- What are the remaining unanswered questions about the sustainability of bioenergy?
- How do you assess the further political development of bioenergy in the EU?
- Can you think of any EU policies, both past and upcoming, that are significant for bioenergy production apart from RED?

***Thematic interviews: bio-based plastics***

Introductory question:

- What is your connection to the bioeconomy and bio-based plastics? When and how did you first come into contact with the topic?

**Topics**

**1. EU Bioeconomy Strategy**

Introductory question:

- In your opinion, what should be the primary objectives of promoting the bioeconomy in Europe?

Exploratory question:

- How is the sustainability of biomass addressed in the EU bioeconomy strategy?
- To what degree can European agriculture and forestry satisfy the biomass demand for energy and materials? Where do you see trade-offs that might occur when we substantially increase the demand for biomass for biomaterials and energy in the EU?
- What changes in biomass production and consumption have to happen to transition towards a sustainable and just bioeconomy? What changes in land use?

**2. Contribution of bio-based plastic to sustainability**

Introductory question:

- To what extent can substituting fossil-based plastic with bio-based plastic contribute to the sustainable use of resources in the European Union?

Exploratory questions:

- To what extent can increasing production of bio-based plastics give rise to conflicting sustainability goals? Where are these conflicts of interest?
- To what extent are changes necessary in using (bio-based) plastics (production of raw materials, further processing, consumption, and disposal or recycling)? What regulatory framework conditions need to be created for this?

**3. Single-use Plastics Directive and EU Policy framework on bioplastics**

Introductory question:

- How did the Single-use Plastics Directive and EU Policy framework on bioplastics emerge, and what are their aims?

Exploratory questions:

- In your opinion, to what extent is the production and use of bio-based plastic addressed by the Single-use Plastics Directive and EU Policy framework on bioplastics?
- How were the Single-use Plastics Directive and EU Policy framework on bioplastics discussed in your field? What were the positions of your group? What were the positions of the other groups?
- To what extent have sociatal actors (industry, agriculture, citizens, NGOs, etc.) been involved in shaping the Single-use Plastics Directive and EU Policy framework on bioplastics on bioplastics? What views were expressed by these actors?

**4. Outlook**

- What questions regarding the sustainability of bioplastics have not yet been answered from your perspective?
- How do you assess the further political development of bioplastics in the EU?
